# Supplementary material for: Systematic multiscale models to predict the compressive strength of fly ash-based geopolymer concrete at various mixture proportions and curing regimes
Source: PLoS One. 2021 Jun 14;16(6):e0253006. doi: 10.1371/journal.pone.0253006 (PMC8202944; doi:10.1371/journal.pone.0253006)
Supplement: Attachment — Submitted filename: My reviwer.docx [file pone.0253006.s001.docx]

This paper presents linear, non-linear, and multi-logistic regression models to predict the compressive strength of fly ash-based geopolymer concrete (FA-GP concrete) using 510 mixes collected from the literature. The content is interesting but this reviewer believes there are several critical concerns need to be carried out. Therefore, this reviewer recommends publication of this paper provided that the following major revisions are successfully carried out.

1. The title of this paper does not fit with its content. The title is lengthy and it should be shortened. Please also do not use abbreviations in the title of paper.
2. Author should briefly discuss their innovation in Abstract
3. The authors used the linear, non-linear, and multi-logistic regression models to predict the compressive strength of fly ash-based geopolymer concrete. The authors should clearly explain why they chose these three prediction models in details. Please also explain the advantages and disadvantages of these three prediction models over other prediction models.
4. As authors say: *“In this regard, a comprehensive dataset consists of 510 samples were collected in several academic research studies and analyzed to develop the models”.*

Authors should clearly explain why these 510 mixes are comprehensive enough for their prediction models. Please also explain what criteria have been considered for collecting these 510 mixes from previous studies.

5. Authors should clearly explain on what basis they chose these twelve input variables for the prediction of compressive strength of FA-GP concrete as there are similarities between some input variables
6. Please choose a better abbreviation for fly ash-based geopolymer concrete (e.g. FA-GPC)
7. Introduction is lengthy with unnecessary explanations on geopolymer concrete behavior. Please shorten Introduction and rewrite it with a more focus on literature review on the use of different machine learning methods for prediction of mechanical properties of concrete.
8. In Introduction, please explain the abbreviation for GGBFS.
9. There should be more discussion on results not just providing the results. Please compare the results obtained by the proposed regression models with other machine learning methods such as artificial neural networks, genetic programming or other regressions models and clearly discuss the results.
10. On what basis, 66%, 17% and 17% of datasets were chosen respectively for training, testing and validating? There should be a sensitivity analysis for the discretization of dataset for training, testing and validating. If there is, please clearly discuss the results.
11. As authors say: *“It can be noticed from both figures that the predicted and measured values of compression strength are closer for the NLR mode, which indicates the superior performance of the NLR model compared to other models.”*

Please clearly explain why NRL has a better performance than the two other models.

12. As authors say: *“According to the obtained results, the curing temperature is the most significant variable for the prediction of the compression strength of FBGC for the whole LR, NLR, and MLR models and this is match with a variety of researches that have been performed in the literature”*

Please clearly explain why this happens?

To discuss the importance of curing temperature for compressive strength prediction, please discretize your dataset based on the curing method (ambient and oven curing) and discuss the results for (i) ambient curing only; (ii) oven curing only and (iii) ambient and oven curing as you have already done

13. There are many grammatical errors that need to be corrected
